# Prediction of Risk of Cardiovascular Events in Patients With Stable Angina Using Artificial Intelligence: A Systematic Review

**DOI:** 10.14740/cr2211

**Published:** 2026-08-31

**Authors:** Sunil Kumar, Abdullah Abdul Sami, Manish Kumar, Veena Kumari, Sooraj Kumar, Ashish Shiwlani

**Affiliations:** a Department of Information Technology (IT Project Management), New England College, Henniker, NH, USA; b School of Professional Studies, Northwestern University, Evanston, IL, USA; c Department of Operations and IT Management, Saint Louis University, St. Louis, MO, USA; d Department of Biomedical Engineering, Stevens Institute of Technology, Hoboken, NJ, USA; e Department of Business Analytics, DePaul University, Chicago, IL, USA; f Department of Computer Science, Illinois Institute of Technology, Chicago, IL, USA

**Keywords:** Cardiovascular diseases, Stable angina, Artificial intelligence, Risk prediction, Machine learning

## Abstract

Stable angina pectoris is a prevalent condition with concerning symptoms and an increased risk of myocardial infarction (MI), stroke, heart failure, and mortality. Risk stratification is important in preventive care because the severity of the condition significantly impacts prognosis. With the availability of digital data such as electrocardiogram (ECG) and clinical variables, artificial intelligence (AI) approaches, including machine learning and deep learning, can be beneficial for risk prediction of chronic diseases, such as coronary artery disease (CAD), in patients with stable angina. At the same time, traditional approaches such as Diamond–Forrester, Framingham Risk Score, and PROCAM lag because they rely on linear and population-oriented assumptions. This systematic review, guided by the Preferred Reporting Items for Systematic Reviews and Meta-Analyses (PRISMA) 2020, evaluates AI models that have been developed to predict cardiovascular events in adults suffering from a stable angina condition. Out of 1,250 articles, only five research studies have been included because they predict the risk of cardiovascular diseases in patients suffering from stable angina. Data modalities in these studies include longitudinal electronic health record (EHR) variables, patient-reported outcomes (Seattle Angina Questionnaire), invasive angiography indices (e.g., Gensini score), and 12-lead ECG. With area under the curve (AUC) varying from moderate (about 0.78–0.83) to excellent (> 0.95) based on outcome and data richness, AI enhances discrimination for obstructive CAD and adverse outcomes across studies. This review highlights that there are a limited number of studies on this topic and a lack of clinical validity. Also, the final set of features in model development varies highly. Hence, heterogeneity, a lack of external validation, and implementation limitations exist.

## Introduction

Stable angina, also known as stable ischemic heart disease (IHD), occurs when the myocardial oxygen supply does not meet demand. IHD is one of the major and common causes of cardiovascular illness, disability, and death [1, 2]. American Heart Association (AHA) states that one in three adult Americans suffer from cardiovascular illness, including 16.8 million people with IHD [3, 4]. IHD usually presents as chest pain from physical activity or emotional distress and has an average yearly risk of 3–4% for myocardial infarction (MI) or mortality [5]. Stable angina is a common sign of obstructive coronary artery disease (CAD), which is one of the major causes of heart-related deaths, accounting for approximately one in seven deaths annually in the United States [6]. CAD is linked to adverse cardiovascular events (CVE) with reduced quality of life, and increased healthcare expenses [6].

There is an increased risk of CVE in patients with stable angina. Major CVE occur in 20% of patients with stable angina or IHD by 6 months [7, 8]. In the Reduction of Atherothrombosis for Continued Health registry, just over half of the 26,000 people with established CAD had angina. Patients with stable angina had higher rates of cardiovascular death, heart attack, or stroke over 4 years compared to those without angina (16.3% vs. 14.2%; hazard ratio (HR), 1.19; 95% confidence interval (CI), 1.11–1.27)) [7]. These facts demand a rapid predictive evaluation and management of patients suffering from stable angina to prevent them from ischemic complications leading to chronic CVE, which ultimately affect the quality of life.

Doctors have long used their own judgement or clinical scores like the Framingham Risk Score (FRS) for clinical profiling to assess cardiovascular risk in patients with stable angina [9]. Clinical algorithms such as the Diamond–Forrester algorithm and the PROCAM score estimate the likelihood of CAD using symptoms and risk factors [10, 11]. These risk scores and algorithms have certain limitations. For example, the FRS predicts CVE over a 10-year period, whereas PROCAM is only for MI. They are calibrated differently for different geographical locations, and models may sometimes overestimate the likelihood of obstructive CAD, as in case of FRS [12, 13]. Also, most of these algorithms use clinical variables from symptomatic patients [14]. This is a major limitation highlighting the incorporation of high-resolution physiological data such as electrocardiogram (ECG), and variables depicting lifestyle changes to better predict CVE in patients with stable angina [14, 15].

Patients with stable angina often have noninvasive tests like ECG, which generates a lot of data that traditional models do not fully use [16]. As more digital ECG data and long-term clinical records become available, stable angina is a promising area for the development of artificial intelligence (AI)-based prediction models [16]. In most cases, the clinicians would decipher the ECG output that could cause subjectivity and inter-rater variance. Due to this fact, minor ECG alterations, which are associated with impending heart issues, can be overlooked through normal scoring and human inspection [17]. All these demonstrate that it is necessary to have data-based approaches that integrate both ECG signals and clinical data to enhance the decision on individual risk prognosis [18]. Most AI-ECG digital biomarkers were created to provide prompt evaluation to patients with sudden chest pain, primarily for acute coronary syndrome (ACS) screening [19, 20]. The evidence on the application of these AI tools to non-acute cases is scarce, which leaves a gap in knowledge regarding the ability of algorithms that are trained according to acute ischemia-focused data to achieve high performance in chronic stable patients [21, 22]. Initially, data on AI-ECG biomarkers was obtained to identify and distinguish the degree of acute ischemia, whereas they could be beneficial in identifying and evaluating the severity of CAD in individuals with stable angina [22, 23].

This systematic review summarizes the current literature on the risk prediction of CVE in patients with non-acute or stable angina. This paper provides clinicians and non-clinicians with an overview of AI use in this topic. Also, it highlights that further such studies should be conducted and clinically validated, as this is an important area of research. If a successful model is deployed that can identify the risk of CVE in patients according to their age, lifestyle, and other factors, then several mortalities occurring due to cardiovascular diseases can be reduced. The main objective is to determine the existing evidence regarding AI frameworks in CVE prediction in stable angina patients. The goals of this review are to enable researcher to (1) identify and outline studies using machine learning (ML) and deep learning (DL) to predict cardiovascular risk status in patients with stable angina; (2) assess the datasets that are in use and evaluate their quality; (3) evaluate the predictive ability of current models in cardiovascular risk prediction and examine the application of these models in clinical practice

One surprising aspect of this systematic screening is that out of the 1,250 studies screened, only five unique studies satisfied all the inclusion criteria. The paucity of information is not just a technical constraint of the review but the real situation of the literature available on digital cardiovascular disease research. While the number of papers discussing the use of AI and DL digital biomarkers for triaging and screening ACS has exploded, there is surprisingly sparse evidence available when it comes to chronic stable angina. It will also draw major limitations and shortfalls in the current study and provide future directions. In this way, the goal of the review is to shed light on how AI might be used to enhance personal cardiovascular risk predictions and assist clinicians in making medical decisions for patients with stable angina.

## Methodology

### Articles collection

This systematic review article has been written following the Preferred Reporting Items for Systematic Reviews and Meta-Analyses (PRISMA) 2020 guideline. A rigorous search was conducted with available literature by applying various electronic databases search engines to extract the relevant articles on the prediction of CVE in patients with stable angina using AI. The search databases which we utilized during the search process include PubMed, Scopus, Web of Science and Google Scholar. The choice of databases selected by authors is featured with the need to study both clinical and non-clinical studies, in particular, a combination of cardiovascular studies and AI. The search of peer-reviewed publications was conducted in November 2025, and studies from the last 5 years (i.e., 2020 to 2025) were included. Only research articles were included and review articles, conferences, and short publications like reports, editors, posters, and dissertations were not considered.

### Search strategy

The search strategy included a well-organized search string. To keep the conformity in the search strategy, the same keywords were used across all databases. Search terms were adapted to the specific syntax and indexing systems of each database. The search strategy combined disease-specific terms (e.g., “stable angina,” “chronic coronary syndrome”), AI-related terms (e.g., “artificial intelligence,” “machine learning,” “deep learning”), and outcome-related terms (e.g., “cardiovascular events,” “major adverse cardiovascular events,” “myocardial infarction,” “risk prediction”).

### Eligibility criteria

The eligibility criteria stating the principles on which the articles were included or excluded are shown in Table 1.

**Table 1:** Inclusion and Exclusion Criteria

Category	Inclusion criteria	Exclusion criteria
Population	Adult patients (≥ 18 years) diagnosed with stable angina or chronic coronary syndrome	Patients with acute coronary syndrome, unstable angina, post-MI only cohorts, pediatric populations
Intervention/exposure	Studies applying artificial intelligence, machine learning, or deep learning models	Studies using only traditional statistical risk scores without AI/ML components
Outcomes	Prediction of cardiovascular events (e.g., MACE, MI, mortality, heart failure, stroke, revascularization)	Studies not reporting cardiovascular outcomes or focused only on diagnostic accuracy without outcome prediction
Study design	Observational studies (retrospective or prospective), cohort studies, registry-based studies	Case reports, editorials, letters, commentaries, conference abstracts, posters, dissertations
Data type	Use of clinical data, ECG, imaging, EHR, laboratory data, or multimodal datasets	Animal studies, simulation-only studies, non-clinical datasets
Publication type	Peer-reviewed journal articles	Preprints without peer review, short communications, reviews
Time frame	Published within the last 10 years	Publications which were prior to the specified time window
Language	Articles published in English	Non-English publications

MI: myocardial infarction; EHR: electronic health record; ECG: electrocardiogram; MACE: major adverse cardiovascular events.

### Articles selection

**Figure 1:**
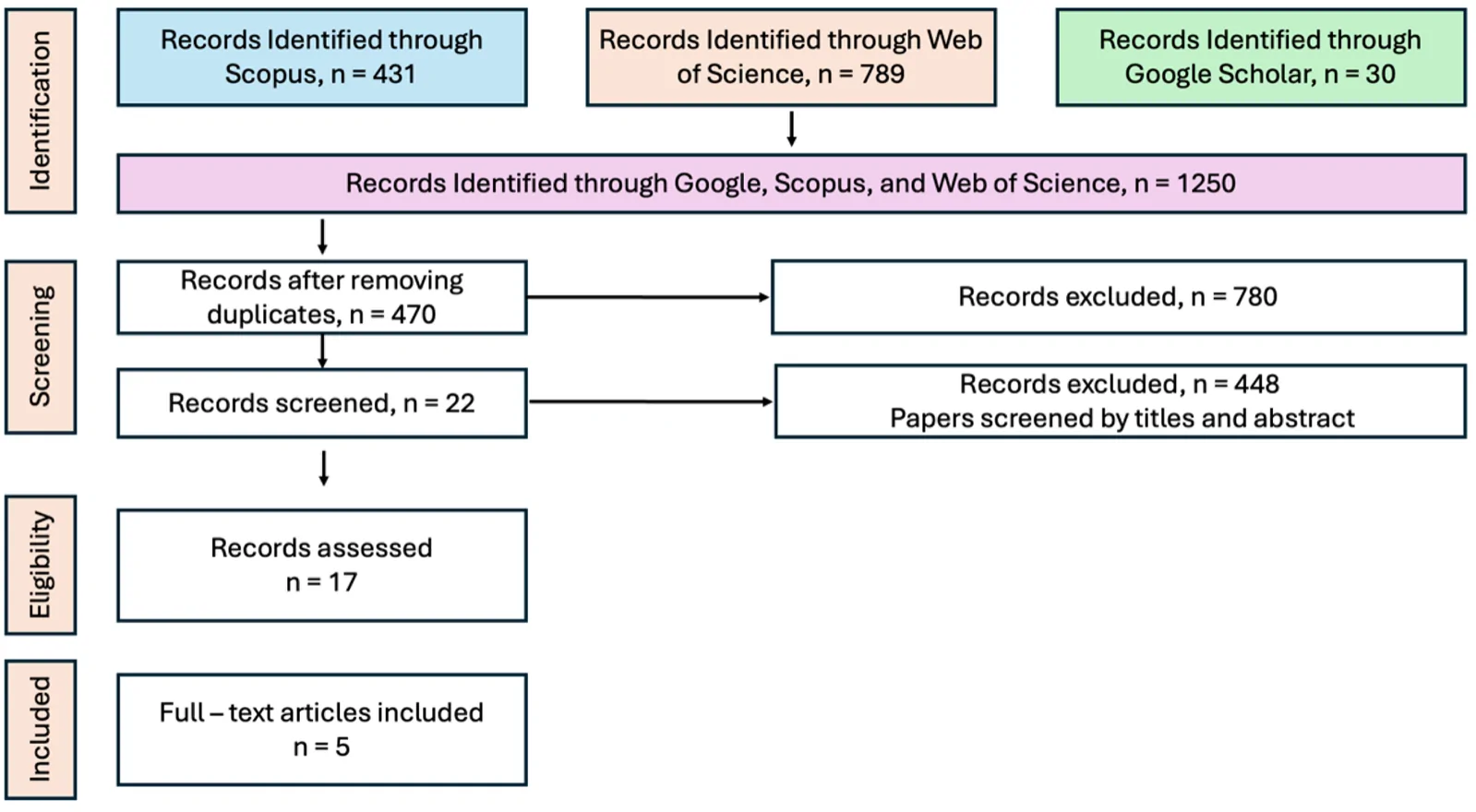
The PRISMA flow diagram for this systematic review. PRISMA: Preferred Reporting Items for Systematic Reviews and Meta-Analyses.

PRISMA 2020 protocol was followed for the inclusion of studies in the systematic review. The first step was identification, and 1,250 records were retrieved from the abovementioned databases. Out of those records, 250 were identified through manual reference search. Then, 780 articles were removed to eliminate duplicate records. A total of 470 articles were assessed according to the inclusion criteria (Table 1). In this screening step of title-based screening, 448 articles were left behind because they did not fall into the research objectives. Only 22 studies were evaluated for abstract screening, and 17 articles were removed during abstract screening due to reasons such as the absence of AI-based prediction models or a lack of relevant cardiovascular outcomes. Following full-text assessment, five studies met all eligibility criteria and were included in the final qualitative synthesis (Table 2) [17–21]. Any disagreements during the screening and eligibility stages were resolved through discussion, with consultation of a third reviewer when necessary. The entire study selection process is illustrated in a PRISMA flow diagram given in Figure 1.

**Table 2:** Summarized Literature for the Systematic Review

Reference	Target	Variable	Architecture	Pre-processing	Dataset	Selection Criteria and Outcome	Result
[17]	AI-ECG detection of obstructive CAD in stable angina	Age, sex, BMI, symptom type, HTN, DM, dyslipidemia, smoking, stroke, FHx + QCG scores + ECG deep features	ResNet-CNN (16 layers, SE blocks, nonlocal); task layers (Adam + focal loss; temp scaling). XGBoost on extracted vectors (80/20; 5-fold CV)	Signal extraction, feature vectors, probability calibration, CV for tuning	SNUBH, 2018–2020, n = 723	Outpatients with stable angina symptoms undergoing invasive angiography. Excluded: asymptomatic, prior CAD/revascularization, and emergency ACS cases.	AUROC: 0.827
						Outcome: any obstructive CAD	
[18]	Obstructive + extensive CAD in suspected stable angina	Clinical risk factors + QCG scores	Modified ResNet encoder	Patient-level split (90/10), bootstrap CI (2000)	SNUBH, 2011–2019, n = 21,866	Outpatients evaluated via invasive angiography/CCTA for chest pain. External validation: independent multi-cohort sample. Excluded: emergency ACS admissions.	AUC: ObstCAD 0.781; severe 0.780; ExtCAD 0.689
						Outcome: obstructive CAD	
[13]	Obstructive CAD and assess prognosis in suspected stable angina	Age, sex, angina typicality, dyspnoa; risk factors (DM, dyslipidemia, FHx); Charlson index	Statistical models: logistic regression (basic + clinical); Cox regression for prognosis	Symptom classification (typical/atypical/non-cardiac), D-F pre-test probability, registry linkage, 30-day blanking period	Single-center, Denmark, n = 3,903	Consecutive single-center outpatient referrals. Baseline status: verified free of baseline CAD and heart failure at entry.	AUC: 0.88
						Outcome: obstructive CAD	
						Composite outcome (death, MI, UA, HF, stroke)	
[19]	12-month CVE in stable angina + CHD	Physical activity, Antiplatelet use, Traditional Chinese medicine, Gensini score, SAQ Exertional capacity, SAQ Anginal stability	ML models: XGBoost, LightGBM, RF, AdaBoost, MLP, SVM, KNN, GNB) + logistic regression,	Prospective cohort, random split 7:3, 10-fold CV, missing MICE imputation, SHAP for interpretability	Single-center, n = 827	Prospective cohort with established WHO/AHA-defined stable coronary disease. Excluded: advanced systemic illness, heart failure, or cognitive barriers.	AUC: train 1.00, validation 0.98, test 0.98 (95% CI 0.97–1.00)
			Best = LightGBM; also kept multivariable logistic regression (nomogram + online calculator)				
						Outcome: composite CVE: nonfatal MI, revascularization, all-cause death, readmission (angina/HF/malignant arrhythmia), stroke	
[20]	ACS risk in stable CHD	EHR vars → QC → 34 clinical vars (labs + diagnoses + utilization + sociodemographics)	T2G-Former + MLP: build feature-relation graph → transformer embeddings → MLP outputs ACS baselines: CatBoost, LightGBM, XGBoost, RF, SVM, FT-Transformer	Convert heterogeneous variables, imputation, patient-level longitudinal mean, MICE (checked vs k-NN), multicollinearity check 5-fold stratified CV, repeated across 12 seeds	Raw: 268,876 CHD records from 165 institutions, n = 12,336	Outpatients with stable angina symptoms undergoing invasive angiography.	The F1 scores were 0.666, 0.757, and 0.886 at 6, 12, and 24 months, respectively.
						Outcome: progression from stable CHD → ACS within 6/12/24 months	

ACS: acute coronary syndrome; AHA: American Heart Association; BMI: body mass index; CAD: coronary artery disease; CCTA: coronary computed tomography angiography; CHD: coronary heart disease; CI: confidence interval; CNN: convolutional neural network; CV: cardiovascular; CVE: cardiovascular events; D-F: Diamond–Forrester; DM: diabetes mellitus; ECG: electrocardiogram; EHR: electronic health records; ExtCAD: extensive coronary artery disease; FHx: family history; HF: heart failure; HTN: hypertension; MI: myocardial infarction; ObstCAD: obstructive coronary artery disease; QCG: quantitative electrocardiography; QC: quality control; UA: unstable angina; vars: variables; WHO: World Health Organization.

## Research Findings

### Characteristics of ongoing research

Due to the high prevalence of cardiovascular disease and its significant morbidity and mortality, preventing recurrent events in patients with established cardiovascular disease remains a critical public health objective. Preventive medicine emphasizes initiating interventions tailored to an individual’s risk level. Consequently, risk assessment is an essential component of preventive care. In 2005, cardiovascular disease accounted for 35% of all deaths in the United States; out of these, 16.7 million were suffering from IHD. This review is analyzing the studies which have been conducted to predict the risk of cardiovascular outcomes in people suffering from stable angina or IHD. It includes five studies published from 2018 to 2025. The studies came from a range of locations, including Korea [17, 18], Denmark [19], and China, with both a single-center cohort [20] and a large multi-institutional electronic health record (EHR) consortium [21]. One study examined mobile-compatible ECG analysis [17], while the others used hospital clinical data [13, 19], or large-scale EHRs [20].

### Types of AI models

AI in stable angina risk prediction primarily employs two approaches: ML and DL. Both approaches identify patterns between clinical factors and cardiovascular outcomes, but they differ in their methods of processing and interpreting clinical data.

#### ML models

ML is a subset of AI. It is a versatile set of techniques or algorithms for finding patterns and relationships in complex data based on selected features and using that information to make independent predictions or decisions. For example, certain clinical and physiological variables can be used to predict a disease risk, similar to how an ML model operates in an autonomous car [21]. The ML models use feature engineering techniques and are usually based on traditional statistical methods. These models enhance their accuracy over time as they are exposed to additional data [21]. In cardiovascular medicine, ML models offer many advantages, including the ability to evaluate enormous volumes of medical data, find hidden patterns, and produce prediction tools applicable across varied patient groups [22].

##### (1) Logistic regression

The logit link function between one binary dependent variable, also referred to as an outcome variable, and one or more independent variables, sometimes referred to as covariates or explanatory factors, is described and estimated by a logistic regression model [23]. It assumes linear predictor to log-odds of the outcome [23]. The use of logistic regression models has been applied to explain events in different medical and nonmedical research fields since they are flexible and well interpreted. More complicated ML models alongside logistic regression is utilized in a Chinese study, which assists in developing risk charts and computing clinical risk [18, 22].

##### (2) Random forest (RF) and k-nearest neighbors

RF is an ensemble algorithm, which uses several decision trees that are suitable in high-dimensional settings where the predictor count can be much higher than the count of observations. It is a nonparametric technique and can deal with any number of outcomes, such as survival time and both quantitative and nominal outcomes. It also can deal with predictors of various scales or distributions [23]. RFs are not used as deployment algorithms but are mostly used as benchmark algorithms in comparative analysis. In addition, they have been employed as their base models in prediction of cardiovascular diseases based on stable angina to select features [19, 22].

##### (3) Support vector machines (SVMs)

SVM is a famous supervised ML method in both classification and regression problems. SVM will attempt to solve a particular binary classification task by being as simplistic as possible, separating the subjects that fall into the two different classes by a classification boundary. This line of classification will be a straight line in two dimensions. SVMs search the best separator hyper-planes amongst groups of high-dimensional data. SVMs are competitive in nature and less efficient in CVE prediction in reviewed studies and demonstrate a trade-off between a reduction of false negative and the preservation of clinical specificity [23]. This weakness limits their independence as clinical tools although they are theoretically sound.

##### (4) Gradient boosting models

Gradient boosting models such as XGBoost and LightGBM are ensemble models that develop more improved predictions with the addition of simple models one after another. Light Gradient Boosting Machine (LGBM) splits trees on a leaf-wise basis, and it takes less time to compute; XGBoost splits trees on a level or depth-wise basis. LGBM is susceptible to overfitting in the implementation process of the model due to the splitting down of leaves, but it can reduce loss and maximize performance. They are usable with numerous organized clinical information. The gradient boosting models are also compared in the reviewed studies with other methods of ML and tend to perform better [23]. To illustrate, in ECG-related works, XGBoost is contrasted with either ML or DL models, and it is determined that the former can be useful in supplementing the latter learning features [23].

#### DL models

DL is the discovery of intricate patterns based on raw or intricate data learning. Such factors as manually selected features are not needed with ML, whereas they are particularly useful in identifying hidden or complex disease signals [23].

##### (1) Convolutional neural networks (CNNs)

CNNs are data pattern learners such as images or signals. CNNs are currently frequently employed to interpret ECGs and identify the invisible disease in heart medicine. In a study using ECG as inputs, a ResNet CNN using special attention blocks is used to analyze images of 12-lead ECG of patients with stable angina [23]. The model is based on the idea that heart ischemia causes electrical changes that may not be obvious in standard tests but still hold important information. This shows that ECG-based DL can find blocked arteries and high-risk heart anatomy that standard ECG readings might miss.

##### (2) Transformer-based models

Transformers easily achieve rapid learning by focusing on long sequences, where weight matrices are used to hold the data that needs to be gradually learnt across millions of training steps. Information stored in long-term memory as key-value pairs can be retrieved by a transformer by constructing a query that considers that information [20]. They use attention mechanisms to show how different features relate to each other. Despite being relatively new to the ML scene, transformers have quickly emerged as the pinnacle of innovation in the field of natural language processing. They are essential in modern AI applications due to their inherent capacity to handle complex sequential data using creative network architecture components [20]. The transformer model predicts the shift from stable heart disease to ACS better than other models, across different time periods.

### Datasets

The datasets that were used in these studies for the prediction of CVE from patients suffering from stable angina include SNUBH (2011–2019), SNUBH (2018–2020), Danish National Patient Dataset, China–Japan Friendship Hospital from October 2020 to October 2021, and National Heart Disease Database Consortium. The cumulative data infrastructure across the reviewed literature spans five distinct cohorts, ranging from localized institutional samples (n = 723 [17]; n = 827 [19]; and n = 3,903 [13]) to large-scale registry populations (n = 21,866 for the 2011–2019 SNUBH cohort [18] and a multi-institutional EHR footprint of n = 12,336 refined from 268,876 raw coronary heart disease (CHD) records [20]). These datasets include symptomatic patients suffering from stable angina with available medical records including clinical risk factors, baseline examinations including blood tests, ECG, and echocardiography. Age, sex, hypertension, diabetes mellitus, dyslipidemia, smoking status, and prior cerebrovascular disease are examples of core clinical and demographic variables that are regularly included, reflecting their established role in IHD risk stratification. The degree to which the studies describe patient status and disease burden, however, varies significantly. While angiography-centered and EHR-based datasets include anatomical severity (e.g., number of diseased vessels, Gensini score), laboratory biomarkers, medication exposure, and healthcare utilization patterns, ECG-based datasets priorities electrophysiological signals as primary inputs and rely less on clinical covariates. The symptoms, follow-up durations, event-rate and patient-reported outcomes (PROs), including angina typicality and Seattle Angina Questionnaire (SAQ) domains, are worth mentioning as a strength factor in some cohort, as they reflect the dimensions of functional impairment and the quality of life which are often under-reported when using traditional risk modeling [13, 22].

The AI model maturity can be seen in the vast differences in the scale of data sets and the number of instances in the studies. Single-center angiography and ECG-based [19] studies of hundreds or thousands of patients permit phenotyping and are limited when generalizations are required and more prone to selection bias, particularly in studies defined by greater enrichment with patients who are invasively tested. Its large multi-institutional EHR dataset, in turn, has great statistical power and population-wide coverage that accounts for more than 12,336 refined patient records that were based on more than 268,876 raw entries [20]. Nevertheless, such a scale is also associated with certain shortcomings, such as a large rate of missingness or the lack of consistent data-gathering procedures across the institutions and reliance on imputation procedures that might conceal clinically relevant time-varying patterns. Although complex methods such as sensitivity analysis and longitudinal MICE imputation address some of the concerns, such crucial variables as imaging outcomes, ECG morphology, and symptom severity are all either not available or not adequately represented in EHR-only data. Moreover, even large sample sizes do not eliminate the issue of outcome imbalance, particularly in the near-term, leading to weighted loss functions, and the question of whether calibration of outcomes can be done in a real-world. The future progress would be useful to standardize variable definitions, multimodal data combination (ECG, imaging, labs, and PROs), prospective data collection, and external validation of AI-driven risk prediction in stable angina across the different healthcare settings, despite the current datasets demonstrating impressive coverage and technical complexity.

### Predicted outcomes

AI models were mainly used for the prediction of major adverse cardiovascular events (MACEs) in stable angina patients. These models were able to identify the high-risk subgroups in stable angina patients using variables indicating anatomical severity, symptom burden, and systemic biomarkers. These results support the idea that stable angina is a dynamic risk state that can be continuously assessed by data-driven modeling. MACEs are a composite endpoint that is widely used to assess the overall risk of cardiovascular disease, which typically includes cardiovascular death, MI, stroke, and heart failure hospitalization [22]. In patients with stable angina, MACE is a marker of long-term disease progression rather than acute instability. Evidence from a large cohort study of 11,223 patients with stable angina showed that the risk of MACE progressively increased with the severity of CAD. Compared with a reference population, patients with normal coronary arteries had a 52% higher risk of MACE (HR = 1.52; 95% CI, 1.27–1.83), whereas those with diffuse non-obstructive CAD had an 85% higher risk (HR = 1.85; 95% CI, 1.51–2.28) after adjustment for traditional cardiovascular risk factors.

### Predictive performance and clinical reliability

Across all the studies reviewed, AI-based models demonstrated a strong ability to distinguish between outcomes and often outperformed traditional risk prediction methods. The reported values of area under the curve (AUC) were moderate (0.78) in the context of ECG detection of obstructive CAD [17] to very high in the context of determining future CVE (more than 0.95) [17] and large-scale ACS prediction with EHR [20]. There was a balance of sensitivity and specificity, in general. High-sensitivity transformer-based models align with the clinical aim of ensuring that high-risk patients are not missed [20].

Formally, calibration (the relative accuracy of predicted risks being accurate) was verified by prospective studies in calibration plots and with decision curves. These techniques were clinically reliable [19]. But the patient cohort is small, and it can also denote overfitting especially when the AUC scores are 1.00 for training and 0.98 for testing. Although there is a shortage of independent external validation, cross-validation, internal test sets, and data in various institutions were used to aid in making sure that the studies were robust [20].

Generally, the analysis demonstrates that AI-based models can differentiate the outcomes, but they are not comparable due to the substantial heterogeneity in datasets. ECG models have average detectivity of obstructive CAD, whilst those that incorporate additional clinical or long-term EHR information tend to have high AUCs, usually greater than 0.90 [17, 19, 20]. Notably, the sensitivity optimization is highlighted in a number of studies, reflecting the clinical priority of minimizing high-risk patients missed in stable populations [20]. Calibration and decision-curve studies identify the compliance of AI predictions with observed results, which justify possible clinical use [19].

In papers analyzed, the conventional risk models or pre-test probability models such as the Diamond–Forester are prone to overestimate the risk of obstructive coronary disease and fail to forecast subsequent CVE in patients of stable angina [23]. Algorithms based on AI would always obtain an additional predictive relevance to clinical variables by themselves and enhance discrimination and risk reclassification [17, 19, 20]. The facts indicate that AI must not be considered as a replacement of clinical judgment but be used as a decision-support tool, where it becomes better to identify hidden risk patterns in various forms of data.

## Discussion, Limitations and Future Directions

Stable angina (traditionally defined in the context of chronic coronary syndrome) is conventionally defined as predictable symptoms (associated with exertion) due to a balance between supply and demand mismatch in myocardial oxygenation. But not to be unstable is not to be benign. Not only is it a clinical issue of recognizing obstructive CAD, but it is also a clinical issue of determining the patients, despite their outpatient stability, who will report adverse events in the near to mid-term. These studies are the ones that demonstrate that AI is at this crossroad: it quantifies multi-domain risk (anatomic, physiological, symptomatic, systemic) into individualized estimates of probability that can be used to make preventative decisions [16, 20].

One of the common themes across the reviewed studies is that stable angina risk exists on a continuum rather than a dichotomy. The modeling of the Danish cohort shows that graded risk separation is also clinically relevant even with small event rates: predicted probability strata are associated with future composite outcomes, and it is important to note that even stable presentations have a significant amount of prognostic heterogeneity [13]. This agrees with the modern theory that chronic coronary disease is a manifestation of persistent atherosclerotic activity, endothelial dysfunction, microvascular dysfunction, and systemic inflammation processes, which vary over time and interact with the burden of comorbidity, treatment effects, and functional status. Nonlinear interaction AI models, particularly those capable of learning, are well suited to match this biological reality since such models do not expect risk factors to affect the outcomes in a merely additive or linear fashion.

The performance and clinical relevance of AI are directly proportional to the richness of the input modality across studies. ECG-based methods consider the resting 12-lead ECG to be a high-resolution physiological sensor. In the latter, deep convolutional networks (e.g., ResNet variants) are trained on latent electrical signals that could represent ischemia-induced conduction alterations, ventricular remodeling, or diffuse subclinical injury that is hard to identify by the eye or handcrafted features [17]. The implication is significant: in the case of stable ischemic risk, which induces subtle, distributed patterns of resting electrical activity, the ECG is a scalable screening medium in outpatient risk stratification, particularly when it is paired with mobile-compatible acquisition and deployment pathways [17].

Conversely, angiography-based cohorts incorporate anatomical disease burden directly via any of a variety of structured measures (vessel disease or Gensini score). The potential Chinese cohort shows that the angiographic severity with the event prediction of symptom burden (SAQ domains) and medication exposure are highly discriminative at 12 months [19]. In principle, this is a more explicit multi-axis definition of chronic coronary disease: anatomy is used to measure the severity of substrates, SAQ is used to measure both the functional and symptomatic manifestations, and treatment factors are used to estimate the intensity of secondary prevention. Models constructed based on these integrative domains may be clinically convincing since their drivers can be directly interpreted and relate to actionable interventions.

The EHR-based models take this reasoning a step further, and model disease as a longitudinal system: lab pathways, diagnoses, utilization, and sociodemographic background all bring the dynamic state of physiology and healthcare interaction pattern of the patient closer to approximation. The multi-institutional, transformer-based study, which is large, demonstrates that discrimination to progress to ACS is high at 6–24 months in case EHR variables are designed, and feature relationships are explicitly modeled [20]. This helps to make an important argument about the management of stable angina: prediction windows should not only be statistically powerful, but also meaningful in clinical terms. A high-risk model within a time period, during which clinicians can escalate therapy, increase vigilance, or increase testing, has more operational utility than a model with a higher AUC, but with a time span that is longer.

The evidence reviewed demonstrates that AI in stable angina tends to take two complementary directions: interpretable modeling and high-capacity modeling. Logistic regression and clinically based stratification models are examples of approaches that can be interpreted; they can be attractive when such factors as clinician confidence, congruence with guidelines, and ease of implementation are considered [13, 19]. The prognosis of the Danish study (logistic regression and Cox regression) demonstrates that classical modelling can still be useful in conducting risk stratification and benchmarking of modern cohorts (younger) against older pre-test probability assumptions [13]. These do not mean that they are AI in the DL sense, but they still belong to the ecosystem of predictive and are frequently used as strong baselines in medical AI development.

High-capacity models, such as CNNs on ECG and transformer encoders on tabular EHR, focus on the representation learning, which encodes delicate patterns, feature interactions that go beyond manual feature engineering [17, 20]. Notably, the studied works do not represent DL as a complete substitution of classical modeling. Rather, they usually apply comparative pipelines: several algorithms are tested, calibration and clinical usefulness are measured, and a good model is chosen considering further use [19, 20]. This is an attitude of pragmatic, decision-support: the best model is the one that works well, generalizes across populations, and is explainable/integrable into workflow, not necessarily the most elaborate.

The targets foreseen in the scrutinized studies are (1) obstructive CAD detection; (2) composite CVE (CVE/MACE-like endpoints); and (3) stable CHD-to-ACS progression. These are connected yet not synonymous. Detection of obstructive CAD is primarily diagnostic; it aids in decisions about testing and revascularization. However, it does not necessarily predict risky events due to plaque mechanisms of stability, systemic inflammation, and response to therapy, not just the severity of the stenosis. The preceding ECG work is moderately-strongly discriminating against obstructive CAD and high-risk anatomy, making it possible to triage patients with AI-ECG to subsequent examination [17]. Other ECG development/validation works indicate similar moderate discrimination across obstructive/extensive phenotype [18].

Event prediction (12-month CVE, 624 months ACS progression), on the other hand, is prognostic and has closer goals with preventive cardiology. Prospective cohort model that combines Gensini and SAQ domains has very high AUCs, and the EHR transformer model is able to maintain high AUC/F1 and sensitivity profiles over time windows [19, 20]. The clinical significance of these outcomes is that they project to the escalation decisions: optimality of antiplatelet/lipid therapy, referral advantage of imaging, the strength of follow-up, and patient education of warning symptoms.

The issue of whether a model can predict is always a steady obstacle to implementation in chronic disease, but the question is whether clinicians can make the case to act on it. Two of them specifically introduce interpretability into the modeling story: SHAP-based explanations to a transformer model and nomogram/risk calculator representation to a regression-based model [19, 20]. This is more than a cosmetic consideration. Explainability links model predictions to mechanistic accounts that are familiar to clinicians: anatomical disease severity, lipid-related biomarkers, indicators of inflammation/coagulation, and functional impairment are all plausible contributors to adverse events. In case interpretability finds common key drivers between horizons (e.g., cholesterol-related and systemic biomarkers in EHR-based prediction), the former enhances face validity and may facilitate shared decision-making [20]. Similarly, model scores can be converted into bedside tools using nomograms and online calculators to facilitate their use in outpatient clinics (when managing the long-term management of stable angina) [19].

### Limitations

The included studies varied significantly in the definition of the population (suspected vs confirmed CAD, stable angina vs stable CHD), results (obstructive CAD vs CVE vs ACS progression), and input modalities (ECG images, angiography + SAQ, EHR). This heterogeneity is a realistic practice but does not allow quantitative pooling and makes cross-study comparison of performance measures difficult [13, 17–20]. The values of AUC, in specific cases, are not directly comparable because of differences in disease prevalence, endpoint definitions, and follow-up horizons.

Despite EHR study merging multi-institutional data and using strong internal validation techniques, most of the models continue to be developed within small geographical and healthcare-system settings, and external validation across geographies, device ecosystems, and practices is not always presented [21–23]. In the case of stable angina, where the risk of baseline, the intensity of treatment, and patient referral patterns are variable, transportability is very critical.

Significant signal may be indicated by very high AUCs (near 1.0) in certain cohorts, though it also creates issues in terms of events, feature leakage, outcome adjudication consistency, or cohort enrichment [21]. Despite this cross-validation and scrupulous imputation, performance inflation may occur when models implicitly learn site-specific patterns of practice (such as testing intensity and admission thresholds) rather than pathophysiology.

Large datasets of EHRs have limitations inherent to them: inconsistent testing, uneven coding, and massive missingness that must be filled in. Even though more advanced imputation (e.g., MICE; sensitivity checks against k-NN) makes imputation more robust, imputation may regularize clinically significant extreme cases and hide temporal degeneration patterns [18]. Smaller single centers, on the other hand, can be more faithful but less representative and more susceptible to selection bias (usually enriched with invasive angiography) [17, 19].

In addition to the problem of sample variance, the problem of a lack of fine-grained data on secondary prevention drugs, such as antiplatelets, statins, and β-blockers, is widespread. Only one study [19] specifically addressed the presence of an antiplatelet drug within the model framework. Other research either did not include any parameters about medication at all [17, 18] or used wide exposure classes obtained through EHR information [18] without tracking important factors like frequency, dosage, and medication adherence. Such absence of fine-grained data about medications is a critical deficiency of the existing clinical AI model design because it limits the reliability of risk predictions.

In the literature, the problem of algorithmic bias based on sex, age, socioeconomic status, and care-access patterns is not always predetermined. They are essential in stable angina, where referral pathways and symptom presentation vary by sex and comorbidity, and referral-enhanced cohort-trained models might perform poorly in primary care. Besides this, most of the studies do not pay enough attention to the issue of governance: monitoring drift, recalibration schedules, liability, and regulatory preparedness to implementation in practice.

Future directions

This risk stratification includes potential deployment in which AI outputs are clinically validated first. Clinical reasoning is best formed in models that combine domains that are complementary: ECG models physiology, imaging/angiography models anatomy, SAQ models symptoms/function, and EHR models systemic trajectory and patterns of care [22]. Future models must focus on multimodal fusion with clear ablation studies that show increases in value with each modality [23]. Further research must regularly survey performance according to sex, age categories, comorbidity load, and socioeconomic proxies, particularly since the pathways to diagnose stable angina are different in even the subgroups [22]. They should also report calibration-in-the-large and calibration slope to be used in clinical decision-making [23].

Privacy-preserving analytics and federated learning would allow the training to be performed on the cohort with geographical diversity without centralization [17]. This is especially pertinent to sensitive ECG/EHR data and would enhance model robustness across healthcare systems characterized by diverse practice styles. In addition to predicting risk, the models must be connected to action: how should they change when the risk is high? Clinical pathways (e.g., more aggressive lipid therapy, more aggressive follow-up, more aggressive imaging) should be defined in the future, strategies should be implemented to define what enhances the use of tests, and the evidence-based approach in the future should be whether AI-directed care can reduce events, decrease costs, or decrease the time to intervention [23].

## Conclusions

A common ailment with worrisome symptoms, stable angina pectoris raises the risk of MI, stroke, heart failure, and death. Because prognosis is greatly impacted by the severity of the ailment, risk stratification is crucial in preventative care. AI techniques, such as ML and DL, can be helpful for predicting the risk of chronic diseases, including CAD, in patients with stable angina, since digital data, such as ECGs and clinical factors, are readily available. However, because of their reliance on linear and population-oriented assumptions, classic methods such as Diamond–Forrester, Framingham Risk Score, and PROCAM lag. Five recent studies are summarized in this systematic review to show how AI-based models improve cardiovascular risk assessment in stable angina and stable CHD by utilizing data that traditional scores underutilize, specifically ECG morphology, angiographic severity, patient-reported symptom burden, and longitudinal EHR trajectories. The evidence is still limited by study heterogeneity, a lack of external and prospective validation, and inconsistent outcome definitions, even with impressive, reported performance. While future multicenter, prospective deployments with standardized reporting are necessary before widespread adoption in stable angina care, AI generally seems most prepared to serve as decision-support enhancing, not replacing, clinical reasoning.

## Data Availability

The authors declare that the data supporting the findings of this study are available within the article.

## References

[1] Bairey Merz CN, Pepine CJ, Walsh MN, Fleg JL (2017). Ischemia and No Obstructive Coronary Artery Disease (INOCA): developing evidence-based therapies and research agenda for the next decade. Circulation.

[2] Wei J, Cheng S, Bairey Merz CN (2019). Coronary microvascular dysfunction causing cardiac ischemia in women. JAMA.

[3] Neumann FJ (2020). 2019 ESC Guidelines for the diagnosis and management of chronic coronary syndromes: The Task Force for the diagnosis and management of chronic coronary syndromes of the European Society of Cardiology (ESC). Eur Heart J.

[4] Virani SS, Alonso A, Benjamin EJ, Bittencourt MS, Callaway CW, Carson AP, Chamberlain AM (2020). Heart disease and stroke statistics-2020 update: a report from the American Heart Association. Circulation.

[5] Joshi PH, de Lemos JA (2021). Diagnosis and management of stable angina: a review. JAMA.

[6] Blumenthal DM, Howard SE, Searl Como J, O'Keefe SM, Atlas SJ, Horn DM, Wagle NW (2021). Prevalence of angina among primary care patients with coronary artery disease. JAMA Netw Open.

[7] Eisen A, Bhatt DL, Steg PG, Eagle KA, Goto S, Guo J, Smith SC (2016). Angina and future cardiovascular events in stable patients with coronary artery disease: insights from the Reduction of Atherothrombosis for Continued Health (REACH) registry. J Am Heart Assoc.

[8] Hemingway H, McCallum A, Shipley M, Manderbacka K, Martikainen P, Keskimaki I (2006). Incidence and prognostic implications of stable angina pectoris among women and men. JAMA.

[9] Lloyd-Jones DM, Wilson PW, Larson MG, Beiser A, Leip EP, D'Agostino RB, Levy D (2004). Framingham risk score and prediction of lifetime risk for coronary heart disease. Am J Cardiol.

[10] Diamond GA, Forrester JS (1979). Analysis of probability as an aid in the clinical diagnosis of coronary-artery disease. N Engl J Med.

[11] Genders TS, Steyerberg EW, Alkadhi H, Leschka S, Desbiolles L, Nieman K, Galema TW (2011). A clinical prediction rule for the diagnosis of coronary artery disease: validation, updating, and extension. Eur Heart J.

[12] Bittencourt MS, Hulten E, Polonsky TS, Hoffman U, Nasir K, Abbara S, Di Carli M (2016). European Society of Cardiology-recommended coronary artery disease consortium pretest probability scores more accurately predict obstructive coronary disease and cardiovascular events than the diamond and forrester score: the partners registry. Circulation.

[13] Reeh J, Therming CB, Heitmann M, Hojberg S, Sorum C, Bech J, Husum D (2019). Prediction of obstructive coronary artery disease and prognosis in patients with suspected stable angina. Eur Heart J.

[14] Tzoulaki I, Liberopoulos G, Ioannidis JP (2009). Assessment of claims of improved prediction beyond the Framingham risk score. JAMA.

[15] Brindle PM, McConnachie A, Upton MN, Hart CL, Davey Smith G, Watt GC (2005). The accuracy of the Framingham risk-score in different socioeconomic groups: a prospective study. Br J Gen Pract.

[16] Cannon CP, McCabe CH, Stone PH, Rogers WJ, Schactman M, Thompson BW, Pearce DJ (1997). The electrocardiogram predicts one-year outcome of patients with unstable angina and non-Q wave myocardial infarction: results of the TIMI III Registry ECG Ancillary Study. Thrombolysis in Myocardial Ischemia. J Am Coll Cardiol.

[17] Park J, Yoon Y, Cho Y, Kim J (2023). Feasibility of Artificial Intelligence-Based Electrocardiography Analysis for the Prediction of Obstructive Coronary Artery Disease in Patients With Stable Angina: Validation Study. JMIR Cardio.

[18] Park J, Kim J, Kang SH, Lee J, Hong Y, Chang HJ, Cho Y (2024). Artificial intelligence-enhanced electrocardiography analysis as a promising tool for predicting obstructive coronary artery disease in patients with stable angina. Eur Heart J Digit Health.

[19] Wang Z, Sun Z, Yu L, Wang Z, Li L, Lu X (2023). Machine learning-based prediction of composite risk of cardiovascular events in patients with stable angina pectoris combined with coronary heart disease: development and validation of a clinical prediction model for Chinese patients. Front Pharmacol.

[20] Ma H, Bai H, Yan J, Chen Q, Ma Z, Tong S, Zhan Y (2025). AI approaches for predicting progression to acute coronary syndrome among stable coronary heart disease patients. NPJ Cardiovasc Health.

[21] Kufel J, Bargiel-Laczek K, Kocot S, Kozlik M, Bartnikowska W, Janik M, Czogalik L (2023). What is machine learning, artificial neural networks and deep learning? Examples of practical applications in medicine. Diagnostics (Basel).

[22] Luscher TF, Wenzl FA, D'Ascenzo F, Friedman PA, Antoniades C (2024). Artificial intelligence in cardiovascular medicine: clinical applications. Eur Heart J.

[23] Srimaneekarn N, Hayter A, Liu W, Tantipoj C (2022). Binary response analysis using logistic regression in dentistry. Int J Dent.

